# Psychological Empowerment and Work Engagement as Mediating Roles Between Trait Emotional Intelligence and Job Satisfaction

**DOI:** 10.3389/fpsyg.2020.00232

**Published:** 2020-03-06

**Authors:** Yue Gong, Yong Wu, Peng Huang, Xiaofei Yan, Zhengxue Luo

**Affiliations:** ^1^School of Fine Art, Shaanxi Normal University, Xi’an, China; ^2^English Faculty, Zhejing Yuexiu University of Foreign Languages, Shaoxing, China; ^3^Department of Military Medical Psychology, Fourth Military Medical University, Xi’an, China

**Keywords:** trait emotional intelligence, psychological empowerment, work engagement, job satisfaction, occupational well being

## Abstract

The majority of research indicates that trait emotional intelligence (EI) plays a crucial role in personal well being; however, the deeper mechanisms of this link remain unclear. The study explored the impact of psychological empowerment and work engagement in the link between trait EI and job satisfaction. Female nurses (370) completed the EI Scale, the Psychological Empowerment Scale, the Utrecht Work Engagement Scale, and the Brief Index of Affective Job Satisfaction. The results of structural equation modeling demonstrated that work engagement partially mediated the association between trait EI and job satisfaction. Moreover, the serial one mediator model revealed that trait EI could influence job satisfaction via the serial mediating impact of “psychological empowerment–work engagement.” These results help to a better understanding of the association between these variables and demonstrate that high trait EI may improve occupational well being from emotional perspectives.

## Introduction

Creating healthy organizations has become a focal point for organizations in improving employees’ health and well being (Warr, 2007; Di Fabio, 2017). Satisfaction with job has been widely considered as an important element or indicator of personal well being (Warr, 2007; Judge et al., 2017; Mérida-López et al., 2019). Job satisfaction was introduced by Weiss (2002) as a positive and pleasurable evaluative state that an individual makes about his/her job experience, which consists of affective and cognitive components (Judge and Kammeyer-Mueller, 2012; Judge et al., 2017). Moreover, affective job satisfaction and cognitive job satisfaction were considered as the positive experience in a job (Lyubomirsky et al., 2005) and job-related well being (Judge et al., 2002; Rothausen and Henderson, 2019). Therefore, job satisfaction is thought of as a crucial and relevant indicator of subjective well being. Extant literature has demonstrated that both dispositional and situational factors predict satisfaction with job (Hosseinian et al., 2008; Keller and Semmer, 2013; Khany and Tazik, 2015; Mérida-López et al., 2019). This investigation relies on the affective events theory (AET) (Weiss and Cropanzano, 1996) as a framework for elaborating the influence of trait EI on job satisfaction, seeking to explore the deeper mechanism underlying this relationship in one study from emotional perspectives (i.e. work engagement and psychological empowerment).

### Trait Emotional Intelligence and Job Satisfaction

Emotional intelligence (EI) has become an important upsurge in recent years. The two types of EI refer to trait EI and ability EI. Mayer and Salovey (1997) demonstrated that ability EI focuses on the core competencies of to recognizing, processing, and using emotion-laden information, which is measured using a maximum performance test. Trait EI refers to individuals’ perceptions of their emotions, which is assessed with self-report questionnaires (Petrides and Furnham, 2001) and indicates the emotionally related self-perception (Petrides et al., 2007). Our study paid close attention to trait EI assessed via a self-report questionnaire. A substantial body of research has demonstrated that trait EI is essential and important for job-related well being (Zampetakis and Moustakis, 2010; Fu, 2014; Schutte and Loi, 2014; Petrides et al., 2016; Clarke and Mahadi, 2017; Sun et al., 2017). Moreover, a meta-analysis study revealed that EI was positively correlated with job satisfaction (Miao et al., 2016, 2017). Prior studies have demonstrated that the positive influence of EI on job satisfaction is well proven; however, the deeper mechanism of this association is unclear. Thus, we attempted to elucidate the effects of psychological empowerment and work engagement on this mechanism in one study from emotional perspectives on the basis of the AET (Weiss and Cropanzano, 1996).

The AET contributes to elaborating how employees with high trait EI could influence on job satisfaction. AET demonstrates that occurrences with their work may enhance or decrease their positive or negative experience, and our study focuses on motivation and positive experience (psychological empowerment and work engagement) as the interpretive variables for the influence of trait EI on employees’ satisfaction with their job. Motivation and positive experience are important in AET because of their influence on work attitudes (Weiss and Cropanzano, 1996), that is, positive experience (work engagement and psychological empowerment) mediates the association between trait EI and work outcomes (e.g. job satisfaction). Therefore, we assume a mediation model that employees with high trait EI may enhance the perceptions of psychological empowerment and work engagement, which in turn could positively influence job satisfaction.

### Trait Emotional Intelligence, Psychological Empowerment, and Job Satisfaction

Extant literatures showed that psychological empowerment may be a vital mediator between trait EI and job satisfaction. Psychological empowerment represents the motivational construct of an intrinsic task, including four cognitions that reveal a personal orientation: competence, meaning, self-determination, and impact and demonstrates cognitive orientations about their job role (Spreitzer, 1995). Psychological empowerment demonstrated an important motivational resource that may enhance employees’ engagement with their work (Ugwu et al., 2014). Previous researches has revealed that personality was considered an important role in psychological empowerment, such as core self-evaluations (Seibert et al., 2011) and self-esteem (Wang et al., 2013). Thus, we propose that trait EI would act as an important antecedent of psychological empowerment. Research also demonstrated that psychological empowerment plays a positive influence on job satisfaction (Dewettinck and van Ameijde, 2011; Amundsen and Martinsen, 2015; Nikpour, 2018) and have a strong predictive influence on work engagement (Bhatnagar, 2012). A meta-analysis (Seibert et al., 2011) reveals that empowerment is a critical antecedent for work outcomes (e.g. job satisfaction and work innovation). Psychologically empowered followers experience more intrinsic need fulfillment through their job and, thus, acquire more satisfaction with their job. Research also revealed that psychological empowerment act as a mediator of the effect of work context and outcomes (Frazier and Fainshmidt, 2012; Schermuly et al., 2013; Fong and Snape, 2015). On the basis of this reason, we propose that psychological empowerment plays a mediator impact on the association between trait EI and job satisfaction.

### Trait Emotional Intelligence, Work Engagement, and Job Satisfaction

An extensive body of research has revealed that work engagement is considered as a potential mediating variable of the link between trait EI and job satisfaction. Work engagement represents a persistent and pervasive affective–cognitive state that consists of three dimensions: vigor, dedication, and absorption (Schaufeli et al., 2002). Many research have demonstrated that the positive effect of EI on work engagement (Ravichandran et al., 2011; Brunetto et al., 2012; Zhu et al., 2015; Extremera et al., 2018). Moreover, personal resources are crucial antecedent of work engagement on the basis of theory of Job Demands–Resources model (Schaufeli et al., 2002; Bakker and Demerouti, 2014). We explore trait EI, one of the personal resources, which accounted for work engagement. Engaged followers could experience more positive emotions including happiness, satisfaction, and enthusiasm. Several researches have revealed that work engagement is correlated to a positive outcome (Schaufeli and Salanova, 2007; Caesens et al., 2014). Work engagement also was considered as antecedents of job satisfaction (Moura et al., 2014). Studies have also demonstrated that work engagement has acted as a mediator between personality and organizational outcomes, including job performance, career satisfaction, and job (Bakker et al., 2012; Jawahar and Liu, 2016; Ngo and Hui, 2018). In addition, AET posits that dispositions will influence their affective experiences at their work, which in turn, influences job attitudes including job satisfaction (Weiss and Cropanzano, 1996). Thus, we propose that work engagement acts as a mediator on the association between trait EI and job satisfaction.

In summary, the purpose of this study was to investigate the mechanisms underlying the link between trait EI and job satisfaction among Chinese nurses. On the basis of the theoretical rationale above, we expected to demonstrate the following: (1) evidence that psychological empowerment psychological empowerment serves as a mediator between trait EI and job satisfaction and (2) evidence that work engagement mediates the link between trait EI and job satisfaction.

## Materials and Methods

### Participants and Procedure

According to the principle of the academic ethics committee of the Fourth Military Medical University, the sample included 370 (response rate is 96.6%) female participants attending three hospitals in Shaanxi. Nursing occupation was chosen because of their occupational ideals and engagement. Importantly, they help others and do a challenging job. Participants ranged in age between 22 and 46 (*M* = 29.63, SD = 3.74). Paper-and-pencil surveys were administered during the class by the researcher after nurses were provided with necessary information about the study. Participants were voluntary and anonymous and gave a written consent. They completed the measures associated with trait EI, psychological empowerment, work engagement, and job satisfaction.

### Measures

#### Trait Emotional Intelligence

The Wong and Law Emotional Intelligence Scale (WLEIS; Wong and Law, 2002) was used to assess trait EI, which comprises 16 items. The WLEIS contains four subscales: self-emotion appraisals (SEA, e.g. “I really understand what I feel.”), others’ emotion appraisal (OEA, e.g. “I am a good observer of others’ emotions.”), use of emotions (UOE, e.g. “I always tell myself I am a competent person.”), and regulation of emotion (ROE, e.g. “I have good control of my own emotions.”). Each item was assessed on a five-point Likert scale ranging from 1 (strongly disagree) to 5 (strongly agree). This measure has revealed good levels of reliability and validity in different cultures (Wong and Law, 2002; Kafetsios and Zampetakis, 2008; Ishii and Horikawa, 2019). In this study, the Cronbach’s alpha coefficient of SEA, OEA, UOE, and ROE was 0.86, 0.85, 0.84, and 0.90, respectively. The internal consistency of the total WLEIS was 0.91.

#### Psychological Empowerment

Psychological empowerment was assessed with a 12-item scale (Spreitzer, 1995) to assess individual perceptions of psychological empowerment. This scale contains four subscales: meaning (e.g. “The work I do is meaningful to me.”), competence (e.g. “I have the skills necessary for my job.”), self-determination (e.g. “I can decide on my own how to go about doing my work.”), and impact (e.g. “I have significant influence over what happens in my department.”). Participants responded on a five-point Likert scale ranging from 1 (strongly disagree) to 5 (strongly agree). This measure has revealed good levels of reliability and validity (Joo and Lim, 2013; Amundsen and Martinsen, 2015; Dust et al., 2018). In this study, the Cronbach’s alpha coefficient of self-determination, competence, meaning, and impact were 0.86, 0.85, 0.84, and 0.93, respectively. The internal consistency of the total psychological empowerment was 0.90.

#### Work Engagement

Work engagement was assessed with the Utrecht Work Engagement Scale (UWES-9) (Schaufeli et al., 2006). The UWES-9 covers three dimensions: vigor (VI, e.g. “at my work, I feel that I am bursting with energy.”), dedication (DE, e.g. “I am proud of the work that I do.”), and absorption (AB, e.g. “I get carried away when I am working.”). Nurses respond using a seven-point scale from 1 (Never) to 7 (Always every day). This measure has revealed good levels of reliability and validity (Gutermann et al., 2017; Van Wingerden et al., 2017). In this study, the Cronbach’s alpha coefficient of VI, DE, and AB were 0.61, 0.79, and 0.82, respectively. The internal consistency of the total WE was 0.89.

#### Job Satisfaction

Job satisfaction was assessed using a Brief Index of Affective Job Satisfaction (BIAJS) questionnaire, a four-item measure of affective job satisfaction (Thompson and Phua, 2012). All items were rated on a five-point scale ranging from 1 (Strongly disagree) to 5 (Strongly agree). A sample item was, “Most days, I am enthusiastic about my job.” This scale has revealed a good reliability and validity (Hirschi, 2014). In this study, the internal consistency of BIAJS was 0.87.

### Analytical Procedure

A two-step procedure was adopted to analyze the mediation effects (Anderson and Gerbing, 1988). The measurement model was tested to estimate the extent to which each latent variable was represented by its indicators. Then, the structural model was examined if the measurement model was accepted, using the maximum-likelihood estimation in Mplus7.4. The following several indices were adopted to assess the overall fit of the model (Hu and Bentler, 1999; Kline, 2011): chi-square statistic (χ^2^), df, the standardized root mean square residual (SRMR) ≤ 0.08, the root mean square error of approximation (RMSEA) ≤ 0.08, the comparative fit index (CFI) ≥ 0.90, and the Tucker–Lewis index (TLI) ≥ 0.90, revealing a good fit.

## Results

### Descriptive Statistics

Table 1 displays the intercorrelations among the study variables and indicates that all variables are highly positively associated with one another.

**TABLE 1:** Descriptive statistics and Pearson correlations among study variables (*N* = 370).

	*M*	SD	1	2	3	4
1TEI	4.05	0.47				
2PE	4.17	0.54	0.56**			
3WE	6.02	0.88	0.44**	0.51**		
4JS	3.95	0.67	0.46**	0.42**	0.56**	

**p < 0.01. TEI, trait emotional intelligence; PE, psychological empowerment; WE, work engagement; JS, job satisfaction.

### Measurement Model

There are four latent factor (trait EI, psychological empowerment, work engagement, and job satisfaction) and 15 observed variables in the measurement model. Four models are compared to distinguish study variables including trait EI, psychological empowerment, work engagement, and job satisfaction using CFA. We compared one-factor model (all observed variables loaded on a single factor), two-factor model (trait EI and psychological empowerment as one factor and work engagement and job satisfaction as the other), three-factor model (trait EI and psychological empowerment as one factor and work engagement and job satisfaction as separate factors), and four-factor model (trait EI, psychological empowerment, work engagement, and job satisfaction as separate factors). CFA results revealed that the four-factor model demonstrated the better fit as shown in Table 2. This measurement model showed an excellent fit: χ^2^ (84, *N* = 370) = 194.421, *P* < 0.01; RMSEA = 0.060 [90% confidence interval (CI) = 0.049–0.071], CFI = 0.957, TLI = 0.947, and SRMR = 0.041.

**TABLE 2:** Fit indices for the measurement models (*N* = 370).

	χ^2^	df	RMSEA [90% CI]	CFI	TLI	SRMR
One factor	860.605	90	0.152 [0.143, 0.161]	0.702	0.652	0.091
Two factors	581.807	89	0.122 [0.113, 0.132]	0.809	0.775	0.070
Three factors	290.530	87	0.080 [0.070, 0.090]	0.921	0.905	0.050
Four factors	194.421	84	0.060 [0.049, 0.071]	0.957	0.947	0.041

### Structural Model

**FIGURE 1:**
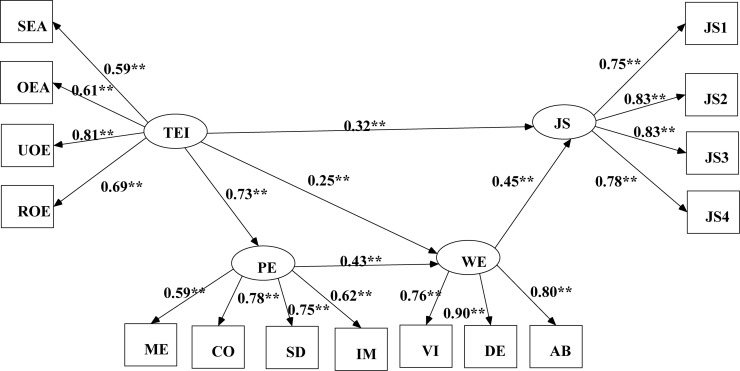
The final model demonstrating effects of trait EI on job satisfaction via psychological empowerment and work engagement. Factor loadings are standardized. SEA, self-emotion appraisals; ROE, regulation of emotion; UOE, use of emotion; OEA, others’ emotion appraisal; ME, meaning; CO, competence; SD, self-determination; IM, impact; VI, vigor; DE, dedication; AB, absorption; JS1–JS4 are four items of job satisfaction. All the path coefficients are significant at the 0.01 level.

Multiple mediation model (Model 1) with two mediators (psychological empowerment and work engagement) indicated a good fit to the data: χ^2^ (85, *N* = 370) = 214.158, *P* < 0.01; RMSEA = 0.064 (90% CI = 0.053–0.075), CFI = 0.950, TLI = 0.938, and SRMR = 0.052. However, the standardized path coefficient from psychological empowerment to job satisfaction was not significant (β = 0.02). Therefore, this path was excluded. Model 2 revealed an adequate fit to the data: χ^2^ (86, *N* = 370) = 214.185, *P* < 0.01; RMSEA = 0.063 (90% CI = 0.053 to 0.074), CFI = 0.950, TLI = 0.939, and SRMR = 0.052. To explore an optimal model, the chain mediation model (Model 3) added one path on the basis of model 2: from psychological empowerment to work engagement. This model fit indexes seemed to be better: χ^2^ (85, *N* = 370) = 194.438, *P* < 0.01; RMSEA = 0.059 (90% CI = 0.048–0.070), CFI = 0.958, TLI = 0.948, and SRMR = 0.041. When comparing Model 3 with Model 2, the results indicated the following: Δχ^2^ = 19.72, Δdf = 1, *P* < 0.01. Therefore, the chain mediation model (Model 3) was proven in our final model (Figure 1). Model 3 revealed that trait EI had indirect effects on job satisfaction, and work engagement partially mediated the relation between trait EI and job satisfaction. In addition, trait EI could influence job satisfaction via the series-mediating impact of “psychological empowerment–work engagement.”

To further investigate the mediation effects, bootstrap analysis was adopted to further investigate the significance of the mediation effects of psychological empowerment and work engagement in the link between trait EI and job satisfaction (Tarrés et al., 2010). Bootstrap samples (5,000) were generated from the original sample set (*N* = 370) through random sampling. The absence of zero in the 95% CI for the estimates indicated that the mediation effects were significant. Table 3 demonstrates the mediating effects of work engagement and psychological empowerment and their associated 95% CIs. As indicated in Table 3, work engagement exerted significant indirect effects on the association between trait EI and job satisfaction. Moreover, the chain-mediating impact of “psychological empowerment–work engagement” in the association trait EI–job satisfaction was significant.

**TABLE 3:** Standardized indirect effects of trait emotional intelligence on job satisfaction through psychological empowerment and work engagement (*N* = 370).

Model pathways	Estimated effects	95% CI	
		
		Lower	Upper
TEI → WE → JS	0.1l^a^	0.03	0.20
TEI → PE → WE → JS	0.14^a^	0.06	0.22

*^*a*^Empirical 95% confidence interval does not overlap with zero. CI, confidence intervals; TEL, trait emotional intelligence; PE, psychological empowerment; WE, work engagement; JS, job satisfaction.*

## Discussion

The main aims of the current study were to investigate the association between trait EI and job satisfaction and examine the mediating effect of psychological empowerment and work engagement in this association. Relying on AET, the results demonstrated that work engagement partially mediated the link between trait EI and job satisfaction, and psychological empowerment do not mediate the association between trait EI and job satisfaction. Moreover, the final model revealed that trait EI could influence job satisfaction via the chain-mediating effect of “psychological empowerment–work engagement.” In summary, these results help to a better understanding of these mechanisms between these variables and indicate that trait EI may impact job satisfaction from emotional perspectives.

The main results are that the indirect impact of trait EI on job satisfaction through work engagement is significant. Work engagement partially and independently mediates the link between trait EI and job satisfaction. In other words, trait EI is associated with job satisfaction via work engagement, which revealed that followers with high trait EI are likely to exhibit greater work engagement and then experience more satisfaction with their jobs. These results are in line with previous studies demonstrating that work engagement is positively associated with job satisfaction and partially mediate the link between proactive personality and job satisfaction (Jawahar and Liu, 2016). Together, these results demonstrate that work engagement has a crucial role in the link between trait EI and job satisfaction. Overall, these findings demonstrated the importance of trait EI in nurses’ work engagement and job satisfaction.

The results demonstrated that the specific indirect impact of trait EI on job satisfaction via psychological empowerment was not supported, which indicates that psychological empowerment does not mediate the association between trait EI and job satisfaction. These findings indicated that to improve employees’ job satisfaction, we should stress increasing their engagement to their work, compared with those solely aiming at improving perceptions of empowerment and demonstrate that the beneficial impact of trait EI on job satisfaction is not simply due to a perception of improving psychological empowerment. Instead, followers with higher trait EI may enhance their job satisfaction through the sequential mediating effect of psychological empowerment and work engagement. In addition, results also demonstrated that trait EI could elaborate job satisfaction via the chain-mediating impact of “psychological empowerment–work engagement.” Hence, followers with higher trait EI could perceive more psychological empowerment and then exhibit more work engagement than those with lower trait EI, and thus contribute to an enhancement satisfaction with their jobs. These findings may be due to the perception that psychological empowerment and showing rich work engagement are quite crucial in enhancing satisfaction with their jobs (Karatepe, 2011; Seibert et al., 2011; Amundsen and Martinsen, 2015; Khany and Tazik, 2015; Jawahar and Liu, 2016; Nikpour, 2018). Taken together, these finds reveal that perception of psychological empowerment and work engagement might be two crucial factors for trait EI followers to enhance occupational well being.

Some limitations of our study should be mentioned. First, causal effects among these variables cannot be concluded due to the cross-sectional approach for our study. Thus, experimental or longitudinal research was adopted to elaborate the causal relationships among these variables. Second, the research relied solely on a sample consisting of nurses working in a hospital and will explore the moderated mediation model of work engagement in this association. Third, our study relies on the total score of trait EI, empowerment, or engagement. Future research will investigate the facts of these variables in these associations. Fourth, future research will explore the other different personal characteristics (such as proactive personality) in trait EI and job satisfaction.

## Conclusion

Our study contributes a deeper understanding of the association between trait EI and job satisfaction and demonstrates that high trait EI may improve occupational well being through the chain-mediating effects of “psychological empowerment–work engagement.”

## Data Availability Statement

The datasets generated for this study are available on request to the corresponding author.

## Ethics Statement

The studies involving human participants were reviewed and approved by the Ethics Committee of the Fourth Military Medical University. The participants provided their written informed consent to participate in this study.

## Author Contributions

XY, ZL, and PH designed the study. YG and XY collected and analyzed the data. YG, YW, and PH wrote the manuscript. ZL supervised the study and edited the draft of the manuscript.

## Conflict of Interest

The authors declare that the research was conducted in the absence of any commercial or financial relationships that could be construed as a potential conflict of interest.
